# The transmembrane channel-like 6 (TMC6) in primary sensory neurons involving thermal sensation via modulating M channels

**DOI:** 10.3389/fphar.2024.1330167

**Published:** 2024-02-19

**Authors:** Yating An, Jingyi Hu, Han Hao, Weixin Zhao, Xiaoxue Zhang, Jicheng Shao, Caixue Wang, Xinmeng Li, Chao Liu, Jinsha He, Yiwen Zhao, Hailin Zhang, Xiaona Du

**Affiliations:** ^1^ Department of Pharmacology, The Key Laboratory of Neural and Vascular Biology, Ministry of Education, The Key Laboratory of New Drug Pharmacology and Toxicology, The Hebei Collaboration Innovation Center for Mechanism, Diagnosis and Treatment of Neurological and Psychiatric Disease, Hebei Medical University, Shijiazhuang, Hebei, China; ^2^ The Forth Hospital of Hebei Medical University, Shijiazhuang, Hebei, China; ^3^ The Key Laboratory of Experimental Animal, Department of Animal Care, Hebei Medical University, Shijiazhuang, Hebei, China

**Keywords:** dorsal root ganglion, TMC6, M channel, heat hyperalgesia, chronic pain

## Abstract

**Introduction:** The transmembrane channel-like (TMC) protein family contains eight members, TMC1–TMC8. Among these members, only TMC1 and TMC2 have been intensively studied. They are expressed in cochlear hair cells and are crucial for auditory sensations. TMC6 and TMC8 contribute to epidermodysplasia verruciformis, and predispose individuals to human papilloma virus. However, the impact of TMC on peripheral sensation pain has not been previously investigated.

**Methods:** RNAscope was employed to detect the distribution of TMC6 mRNA in DRG neurons. Electrophysiological recordings were conducted to investigate the effects of TMC6 on neuronal characteristics and M channel activity. Zn^2+^ indicators were utilized to detect the zinc concentration in DRG tissues and dissociated neurons. A series of behavioural tests were performed to assess thermal and mechanical sensation in mice under both physiological and pathological conditions.

**Results and Discussion:** We demonstrated that TMC6 is mainly expressed in small and medium dorsal root ganglion (DRG) neurons and is involved in peripheral heat nociception. Deletion of TMC6 in DRG neurons hyperpolarizes the resting membrane potential and inhibits neuronal excitability. Additionally, the function of the M channel is enhanced in TMC6 deletion DRG neurons owing to the increased quantity of free zinc in neurons. Indeed, heat and mechanical hyperalgesia in chronic pain are alleviated in TMC6 knockout mice, particularly in the case of heat hyperalgesia. This suggests that TMC6 in the small and medium DRG neurons may be a potential target for chronic pain treatment.

## 1 Introduction

Nociception is a sensory physiological early warning mechanism that is essential for sensing and avoiding contact with noxious stimuli (Woolf, 2010), whereas chronic pain is a debilitating disease characterised by anxiety, insomnia, depression, and other mental illnesses (Pitzer et al., 2019), which is also an unmet clinical problem with vast individual, societal, and economic impacts. Chronic pain conditions feature remarkably in the top ten causes of disability and are the leading cause of disease burden globally (Mills et al., 2019). The pathological activity of the peripheral somatosensory afferents is a major driver of chronic pain. The dorsal root ganglion (DRG) participates in sensory transmission and adjustment, including pain transduction, and carries sensory messages from peripheral receptors to the central nervous system (Esposito et al., 2019). Under pathological conditions, sensory neurons in the DRG generate spontaneous electrical activity, which can be used as a target for chronic pain treatment. Many ion channels, receptors, and signaling proteins in DRG neurons are involved in generating chronic pain (Basbaum et al., 2009; Du et al., 2014; Basso and Altier, 2017; Du et al., 2017; Moran and Szallasi, 2018). Our previous studies demonstrated that voltage gated potassium channel KCNQ2 and KCNQ3 were the predominantly expressed KCNQs in DRG neurons, and formed the heteromeric tetramer called “M channel” (Delmas and Brown, 2005), conducting low-threshold, tardily activating, and non-inactivating currents. KCNQ2 and KCNQ3 channels negatively regulate neuronal resting membrane potential (RMP) and excitability in the DRG and represent important therapeutic targets for chronic pain (Zhang et al., 2011; Du et al., 2014; Taura et al., 2021).

In mammals, the transmembrane channel-like (TMC) family comprises eight members, TMC1–TMC8 (Yue et al., 2019), with approximately ten transmembrane domains and a conserved TMC domain. Members of the TMC family are reportedly associated with several diseases; for instance, TMC8 is a potential biomarker for hepatocellular carcinoma (Lu et al., 2017); TMC5 promotes prostate cancer cell proliferation (Zhang et al., 2019) and is upregulated in pancreatic adenocarcinoma (Gan et al., 2023); and TMC1/TMC2 localising in mouse inner ear hair bundles and converting the mechanical signals of sound into electrical signals, is related to deafness (Jia et al., 2020). Previous studies have reported that TMC is associated with proprioception (Guo et al., 2016; He et al., 2019) and texture discrimination in *Drosophila* sensory systems (Wu et al., 2019). However, the overall contribution of TMC to the development of chronic peripheral pain remains unclear.

In the present study, we found that the TMC6 gene, which is associated with epidermodysplasia verruciformis (EV) (Shimizu et al., 2023), and cervical cancer (Castro et al., 2012) was highly expressed and widely distributed in DRG tissues especially in small and medium neurons (Usoskin et al., 2015). We showed that the TMC6 gene affected mouse thermal behaviour by regulating the neuronal RMP and cell excitability, and restrained the function of the KCNQ channel. After TMC6 knockout, the emergence of more negative RMP, inhibited neuronal excitability, and enhanced KCNQ channel function collectively contributed to the alleviation of peripheral thermal pain sensation. These results suggest that TMC6 may be a new target for analgesia in clinical applications.

## 2 Materials and methods

### 2.1 Experimental animals

Conventional knockout mice of TMC6 gene were purchase from Cyagen Biosciences Company. The mice strain used in this article were C57BL/6. The genotyping strategy that exon 5-exon 10 knockout were applied in the wildtype allele. All the animal experiments were implemented in the Animal Care and Ethical Committee of Hebei Medical University and were in conformity to the International Association for the Study of Pain guidelines for the animal used.

### 2.2 Mouse genotyping

Genomic DNA of mouse could be extracted from 0.5 cm clipped tail specimen by the 100 μL lysed (50 mM NaOH), incubating with a water bath at 95°C for 30 min. Added 30 μL Tris-HCl (1 M, pH = 7.2) per specimen. PCR reaction conditions: 94°C for 3 min; 35 cycles of 94°C for 30 s, 60°C for 35 s, 72°C for 35 s; 72°C for 5 min. The primers of TMC6 were F1: 5′-TTG​TTA​CAA​GAG​TGG​ATT​GGG​TGG-3′, R1: 5′-TAA​AGA​GGC​TCA​GGC​CAT​ACA​TC-3′, R2: 5′-GGT​ATT​AGA​GAG​GAG​GAA​GGG​GAC-3′. The expected product sizes were 585 bp (Homozygotes), 585 bp/450 bp (Heterozygotes), 450 bp (Wildtype allele).

### 2.3 Behavioural experiments

Behavioural tests were performed with male or female mice of TMC6-KO and littermate controls (WT), which were approximately 8-week-old. All the animals put in the test room for 30 min before experiments to acclimate. The experimenters were blinded to the genotype of the mice.

### 2.4 The thermal withdrawal latency

Thermal withdrawal latency was measured using the Hargreaves test (PL-200, Taimeng Co., Chengdu, China). The intensity of the radiant heat source was maintained at 10% ± 0.1%. Mice were placed individually into Plexiglas cubicles positioned on a transparent glass surface. A light beam emitted from a radiant heat lamp situated beneath the glass was directed towards the plantar surface of the hind paw. The time from the onset of radiant heat stimulation to the withdrawal of the hind paw was recorded. To ensure accuracy and reliability, three trials were conducted for each mouse, with a 5 min interval between each trial. The scores obtained from these three trials were then averaged.

### 2.5 Mechanical withdrawal threshold

The mechanical withdrawal threshold was measured using Von Frey test (Stoelting Co.) with a calibrated range of bending force (0.16, 0.4, 0.6, 1, 1.4, 2 g). Each mouse was placed into a plastic cage with a wire mesh bottom. A single filament was applied perpendicularly to the plantar surface of hind paw for a total of five times, with an interval of 5 min between each application. A positive response was defined as at least 3 clear withdrawal responses out of 5 applications. Filaments were applied in an up-and-down order based on the mouse’s response. If there was a negative response, indicating no withdrawal, the next filament with a higher bending force was applied. Conversely, if there was a positive response, indicating withdrawal, the next filament with a lower bending force was used.

### 2.6 Cold/Hot plate test

The mice were placed in a plexiglass bucket on the metal plate precisely set at 2, 46, 48, 50, 52, 54°C. Time of the nociceptive response (clicking the hind paw or jumping) was recorded. To prevent skin scalding, the time of cut-off was 60 s.

### 2.7 Tail-immersion test

The mice were placed in fixators that allowed the tail to move freely. One-third of the tail was immersed in the water bath that set as 46, 48, 50°C. Time of the nociceptive response (flicking the tail) was recorded. To prevent acute tail damage, the time of cut-off was 60 s.

### 2.8 Chronic pain models

The surgery of chronic constriction injury (CCI) as the pathological pain model was performed with aseptic environment. First of all, mice were anesthetized with 0.2 mL 1% Pentobarbital dissolved in saline solution. The mice’s right hind legs were shaved, and sterilized with 75% ethanol. An incision below the femur was made and exposed the sciatic nerve. Four ligatures (4–0 chromic gut) were tied, 1.0–1.5 mm apart, near the sciatic nerve trifurcation. The skin was sutured, and the animal was transferred to a recovery cage. To induce chronic inflammatory pain, complete Freund’s adjuvant (CFA, 25 μL) was injected into the plantar surface of the right hind paw of the mice.

### 2.9 *In vivo* knockdown and overexpression of mTMC6 gene

Knockdown mTMC6 gene of WT mice and re-express TMC6 of TMC6-KO mice in the L4 DRG were achieved by affecting the DRG with AAV9 virus, and were produced from Genechem Co., Ltd (Shanghai, China). The mTMC6 DNA sequence referred to NCBI (GenBank: NM_145439). Vectors construction was GV466 (hSyn promoter-MCS-EGFP-3FLAG-SV40 PolyA). AAV9-mTMC6-cDNA (2.29 × 10^13^ v. g./mL), AAV9-control 323 (1.8 × 10^13^ v. g./mL); AAV9-mTMC6-shRNA (2.33 × 10^13^ v. g./mL), target sequence: CCT​GCA​TCA​TTC​TGG​TAT​A, AAV9-control 533 (1.08 × 10^13^ v. g./mL). Diluted virus to 5 × 10^12^ v. g./mL before using. Mice were anesthetized with 0.2 mL 1% Pentobarbital dissolved in saline solution. The above-mentioned AAV9 virus were injected into the DRG (right L4). Mice were anaesthetized with 1% pentobarbital sodium and ganglions were exposed surgically. Viral solution was diluted with equal volume PBS and injected into the exposed DRG at a rate of 0.2 mL/min (2 mL per DRG) with a glass micropipette connected to a Hamilton syringe controlled by a microsyringe pump controller (78–8,130, KD Scientific, United States). The glass micropipette was removed after 5 min, then the skin was sutured, the animal was transferred to a recovery cage.

### 2.10 Quantitative real-time RT-PCR

The total RNA from L4 DRGs of WT mice that infected AAV9-viruses carrying TMC6-shRNA and control to knockdown TMC6 or TMC6-KO mice that infected AAV9-viruses carrying TMC6-cDNA and control to re-express TMC6 was extracted using a commercial RNA isolation kit (RNAiso, Takara) after chronic pain models behavioural experiments. In this experiment, the isolated RNA was dissolved in 20 μL of diethyl pyrocarbonate (DEPC)-treated water. Then, reverse transcription was carried out using a PrimeScriptRT™ reagent kit from Takara and a thermal cycler (Mastercycler, Eppendorf). For the quantitative PCR reaction, SYBR Premix Ex TaqII from Takara was used. The fluorescent DNA produced during the reaction was detected and quantified using an FQD-48A (A4) system from BIOER. The primers used in standard RT-PCR experiments were: mTMC6-F: 5′-GTC​TTC​CTC​ACC​TTG​CTC​TGC​TTC-3′; mTMC6-R: 5′- GAC​CGT​GCC​TGC​TTC​ATA​CAT​GG-3′.

### 2.11 RNAscope combined with immunofluorescence co-detection

RNAscope (Advanced Cell Diagnostic, ACD; CA) probes targeted mTMC6 (ACD, 1190011-C1) on DRG neuron slides, referring to manufacturer’s workflow. Prepared the fresh frozen DRG neuron tissue sections, embedding DRG neuron tissue into Cryo-embedding medium (OCT) and removed the slide to the −80°C overnight. Then fixed and dehydrated the tissue. Applied Hydrogen Peroxide for 10 min at room temperature (RT). Slides were incubated with primary antibodies NF200 (Sigma, NO142), Periferin (Abcam, ab39374), KCNQ2 (sigma, ZRB1299), KCNQ3 (Abcam, ab302782) at 4°C overnight. Placed slides in 10% neutral buffered formalin (NBF) for 30 min at RT. Added the Protease III to each section for 30 min at RT. Slides were hybridized with the RNAscope probes targeting mTMC6 and incubated in the pre-warmed HybEZ oven for 2.5 h at 40°C. The RNA of TMC6 signals were amplified with TSA Plus fluorophores and incubated in the pre-warmed HybEZ oven for 30 min at 40°C. Stained Secondary antibody for 30 min at RT. Finally, the slides were redyed with DAPI and mounted with coverslip.

### 2.12 Exogenous expression system

The HEK293 cell lines (Kunming Cell Bank, KCB2000408YJ) have been widely utilized in fundamental ion channel research due to their ease of cultivation, efficient transfection, and expression capabilities. Initially, HEK293 cells were seeded into a 24-well plate. When the cell density reached 80%–90% the following day, the cells were transfected with 500 ng/plasmid of pReceiver-CMV-TMC6-GFP, pcDNA3.1-CMV-KCNQ2, and pcDNA3.1-CMV-KCNQ3 using 0.5 μL/plasmid of DNA transfection reagent (Neofect, TF20121201) diluted with 50 μL/plasmid of Opti-MEM (Thermo, 31985-070). The mixed reagents were left to stand for 30 min at RT before being added to one well of the 24-well plate. On the third day, the cells were digested and transferred onto a 10 mm microscope cover glass, which could then be used for electrophysiological recordings.

### 2.13 Electrophysiological recordings

Whole‐cell patch‐clamp recorded with thin-walled borosilicate glass capillaries that were pulled as electrodes (resistance 3–5 MΩ), both on HEK293 cells transfected with the desired plasmids and mouse DRG neurons. The pipette was filled with solution containing (in mM): 140 KCl, 1 MgCl_2_, 5 EGTA, 10 HEPES, pH 7.2–7.4 adjusted with KOH. The extracellular solution contained the following (in mM): 140 NaCl, 3 KCl, 2 CaCl_2_, 1.5 MgCl_2_, 10 HEPES, and 10 glucose (pH 7.4), pH 7.2–7.4 adjusted with NaOH. The Axon Patch 700B amplifier, Digi-data 1440A system and Clamp 10.0 software were applied in this article. Current-clamp was used for detecting membrane potential and neuronal excitability. The current-clamp’s ramp protocol was from 0 to 1 nA (1,000 ms duration), which measured the firing characteristic of action potential (AP). Voltage-clamp was holding at −60 mV, the one step protocol was single voltage stimulation up to 0 mV, and the multiple-step protocol was voltage stimulation from −100 to 100 mV with gap level of 20 mV.

### 2.14 (6-methoxy-8-quinolyl)-p-toluene-sulfonamide (TSQ) stain

(6-methoxy-8-quinolyl)-p-toluene-sulfonamide (TSQ) stain was used in this experiment. The dorsal root ganglion (DRG) were extracted and immediately transferred to liquid nitrogen for rapid freezing. The DRG tissue was embedded in Tissue-Tek O.C.T. compound (Sakura, United States) and stored at −80°C overnight. The embedded tissue block was cut into 8 μm thick slices using a microtome (Leica, Germany), and the slices were washed with phosphate-buffered saline (PBS). To prepare the working solution for TSQ staining, a diluent containing 0.019 g sodium acetate and 0.029 g sodium barbital was dissolved in 1 mL of water. The resulting solution had a concentration of 0.1 mM TSQ (Keygen Biotech, KGAF027). The slices were then incubated with the TSQ working solution for 30 min, followed by washing with PBS. Images of the stained slices were captured using a Leica confocal microscope system with an excitation wavelength of 334 nm.

### 2.15 FluoZin-3 measurements in cell culture

The DRG neurons were extracted from both WT and TMC6-KO mice and digested using collagenase to obtain individual cells. The cells were then cultured in a 35 mm confocal dish (Biosharp, BS-15-GJM) using DMEM culturing media supplemented with 10% BSA, 1% penicillin and streptomycin mixture. The dish was placed in an incubator set at 37°C with 5% CO_2_. After 2 days, the dishes were washed three times with PBS. The DRG neurons were then incubated with 10 µM FluoZin-3 a.m. (Thermo Fisher, F24195) and 0.02% F-127 (diluted 1:1000) for 30 min at 37°C in the dark. Following the incubation, the cells were washed twice with PBS. Finally, images of the DRG neurons were obtained using the Leica confocal microscope system with an excitation wavelength of 488 nm.

### 2.16 Western blot

The tissue of DRG were dissected with a homogenizer in enhanced RIPA lysis buffer added to the phenylmethylsulfonyl fluoride (PMSF) protease inhibitor, standing for 30 min at ice-cold. The lysis buffer was centrifuged for 25 min at 10,000 rpm and 4°C. The supernatant of the lysate was mixed with ×5 SDS sample buffer for 10 min at 95°C. Proteins were separated by SDS‐PAGE then transferred to membranes of PVDF. Primary antibody rabbit anti-TRPV1 (Abcam, ab305299) was added at a dilution of 1:1000 for overnight at 4°C. Secondary alexa fluor‐conjugated antibodies (Invitrogen) for 2 h at RT. Imaging was performed using Odyssey software.

### 2.17 Statistics

All data were given as mean ± SEM. The statistical analyses were performed with IBM SPSS Statistics 21.0 and OriginPro 2018. Differences between groups were assessed by independent two sample *t*-test (parametric test) or Mann-Whitney U test (nonparametric test). The repeated measurement data were assessed by two-way repeated ANOVA with Tukey post-test. Comparison rates between groups were assessed by Pearson’s chi-square test. Significant difference was considered at *p* < 0.05. * or #, ** or ##, *** or ### indicated significant difference from the appropriate control with *p* < 0.05, *p* < 0.01, or *p* < 0.001.

## 3 Results

### 3.1 The expression pattern of TMC6 mRNA in DRG neurons

**FIGURE 1:**
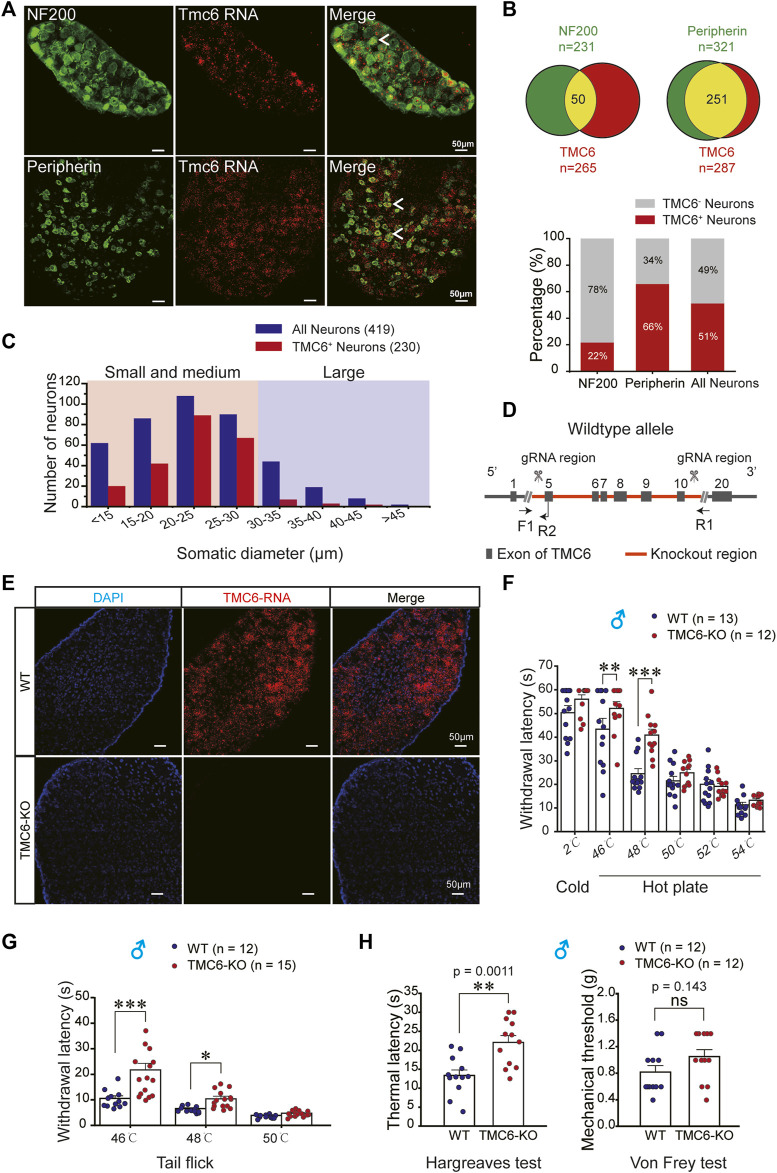
The distribution of TMC6 mRNA in DRG neurons and behavioural effect of TMC6 deletion on thermal and mechanical sensitivities. **(A)** DRG slices stained by TMC6 mRNA using RNAscope probe (red) and antibodies against NF200 (green) and peripherin (green). The co-localization of TMC6 mRNA and the tested protein markers was indicated by arrows. Scale bars, 50 μm. **(B)** The pie diagram illustrates the distribution of DRG neurons expressing NF200 or peripherin (green), and the number of DRG neurons expressing TMC6 (red). The upper panel of the figure demonstrates the co-expression of NF200 or peripherin with TMC6 mRNA, represented by the yellow area. Lower panel provides the percentage of TMC6-positive neurons among NF200-positive, peripherin-positive, or all neurons, respectively. The analyse was obtained from 3 or 4 DRG slices. **(C)** Distribution of TMC6-positive DRG neurons and all DRG neurons across different somatic diameters. **(D)** Diagram of gene knockout strategy for the TMC6-KO mice. **(E)** RNAscope images of TMC6 mRNA in WT and TMC6-KO DRG, TMC6 mRNA (red) and DAPI (blue). Scale bars, 50 μm. **(F)** Withdrawal latencies of TMC6-KO and WT male mice in the cold/hot plate at 2, 46, 48, 50, 52, and 54°C, respectively. WT mice *n* = 13; TMC6-KO mice *n* = 12. **(G)** Withdrawal latencies of TMC6-KO and WT male mice in the tail-flick assays at 46, 48, and 50°C, respectively. WT mice *n* = 12; TMC6-KO mice *n* = 15. **(H)** Baseline thermal withdrawal latencies and mechanical thresholds of the right hind paw were measured in male TMC6-KO mice and their littermate control WT mice using Hargreaves and Von Frey tests. Data are shown as mean ± SEM, two-way repeated ANOVA with the Tukey post-test was used for comparison of behavioural measurements within the corresponding temperature points **(F,G)**, *t*-test or Mann-Whitney U test **(H)**. *p < 0.05, **p < 0.01, ***p < 0.001, ns: no significant.

The expression of TMC6 in the dorsal root ganglion (DRG) of 6–8 week-old wild-type (WT) mice was examined using RNAscope *in situ* hybridization (the positive and negative controls were shown in Supplementary Figure S1). It is shown that TMC6 mRNA partially overlapped with markers for myelinated large neurons (NF200) (Figure 1A, upper panel) and unmyelinated small neurons (peripherin) (Figure 1A, lower panel), which are involved in conveying somatosensory and nociceptive signals, respectively. In Figure 1B, the upper panel (left) displays the results for DRG neurons labelled with NF200 (*n* = 231) and TMC6 (*n* = 265), with 50 neurons co-expressing both TMC6 and NF200. The upper panel (right) displays the results for DRG neurons labelled with peripherin (*n* = 321) and TMC6 (*n* = 287); and 251 neurons co-expressing both TMC6 and peripherin. The lower panel of Figure 1B represents the percentage of TMC6-positive neurons among NF200-positive, peripherin-positive, or all neurons, respectively. We observed that TMC6-positive neurons accounted for up to 66% in peripherin-positive neurons, while only 22% in NF200-positive neurons. The proportion of TMC6-positive neurons among all neurons was 51%. Figure 1C shows the distribution of TMC6-positive neurons across different somatic diameters in the DRG neurons. All of the above result indicated that TMC6 expression was preferentially localized in small and medium DRG neurons.

### 3.2 Generally knocking out of TMC6 affects heat sensitivity in mice

Due to the abundant expression of TMC6 mRNA in DRG neurons, we investigated the potential involvement of TMC6 in peripheral sensory transmission. To this end, we generated TMC6-KO mouse line via knocking out the exon region from E5 to E10 of TMC6 gene (Figure 1D). TMC6 mRNA was abundantly expressed in the DRG neurons of wild-type (WT) mice, but absent in TMC6-KO mice, as determined by RNAscope (Figure 1E). To evaluate the effect of TMC6 deletion on basal thermal and mechanical sensitivities, we performed a series of behavioural tests in WT and TMC6-KO male mice. The results of the cold/hot plate test, as shown in Figure 1F, indicated that TMC6-KO male mice exhibited a significantly elevated thermal threshold at 46°C and 48°C compared to their WT littermates. In the tail flick test, TMC6-KO mice exhibited a pronounced increase in the latency period for the abrupt flailing of their tails, when approximately one-third of the tail was immersed in a water bath at 46°C and 48°C (Figure 1G). The reaction time of both WT and TMC6-KO male mice was dramatically decreased when the temperature exceeds 48°C, and no significant difference was observed between the two groups. Hargreaves and Von Frey tests were then used to detect thermal and mechanical sensitivity of TMC6-KO mice. It was shown that the thermal latency was significantly increased in TMC6*-*KO male mice (Figure 1H, left), whereas there was no obvious change in mechanical threshold compared with WT male mice (Figure 1H, right). Noticeably, TMC6-KO female mice displayed comparable alterations in thermal and mechanical sensitivities, as assessed through Hargreaves and Von Frey tests (Supplementary Figure S2).

**FIGURE 2:**
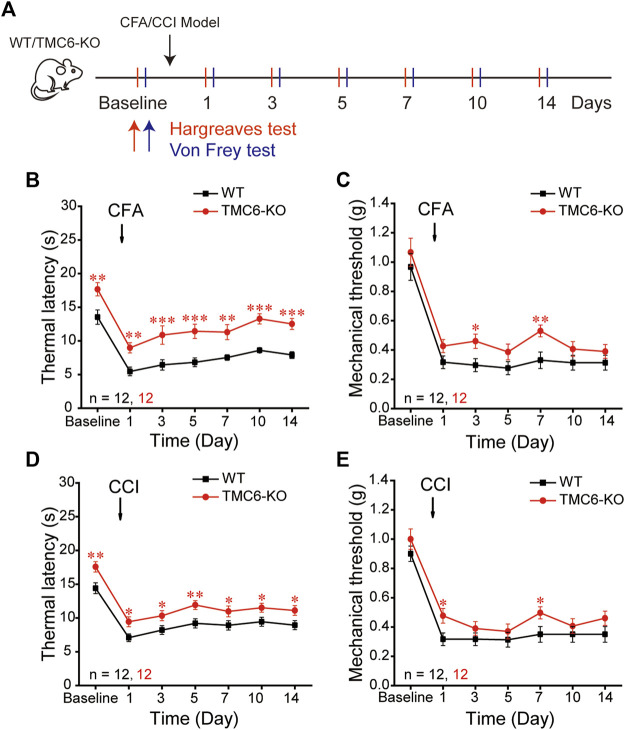
Effects of TMC6 deletion gene on chronic inflammatory or neuropathic pain. **(A)** Schematic diagram of Complete Freund’s adjuvant (CFA, 25 μL) and chronic constriction injury (CCI) model. Hargreaves and Von Frey tests were performed on the baseline and on the 1st, 3rd, 5th, 7th, 10th, 14th days after CFA or CCI operations. A comparison was made between TMC6-KO male mice and their WT littermates regarding chronic inflammatory pain **(B,C)** and neuropathic pain **(D,E)** behaviors. Heat analgesia induced by CFA **(B)** and CCI **(D)** were significantly alleviated in TMC6-KO. The mechanical allodynia was only marginally alleviated in CFA **(C)** and CCI **(E)** models. Data are shown as mean ± SEM. two-way repeated ANOVA with the Tukey post-test was used for comparison of behavioural measurements within the corresponding time points: *p < 0.05, **p < 0.01, ***p < 0.001.

Next, we investigated the effects of TMC6 deletion on chronic pain. We used a chronic constriction injury (CCI) model of neuropathic pain and a peripheral injection of complete Freund’s adjuvant (CFA) as models of chronic inflammatory pain (Jasmin et al., 1998). The thermal and mechanical sensitivities of TMC6-KO male mice and their WT littermates were evaluated on the day prior to CFA injection and CCI surgery, as well as at subsequent time points indicated in Figure 2A. Consistent with the result in Figure 1H, basal thermal latencies of TMC6-KO mice were significantly higher than that of WT mice (Figures 2B, D). In CFA models, thermal hyperalgesia was significantly alleviated in TMC6-KO mice, which showed significant higher thermal latencies on all tested days: 1st, 3rd, 5th, 7th, 10th, and 14th day after CFA injection comparing with WT mice (Figure 2B). However, only the 3rd and 7th days exhibited significantly increased mechanical thresholds in TMC6-KO mice (Figure 2C). Similarly, TMC6-KO mice exhibited a significant alleviation of thermal hyperalgesia compared to WT mice in the CCI models. Significant increases in thermal latencies were observed on the 1st, 3rd, 5th, 7th, 10th, and 14th days after CCI surgery in TMC6-KO mice (Figure 2D). However, only the 1st and 7th days exhibited significantly increased mechanical thresholds in TMC6-KO mice (Figure 2E). All of these results imply that TMC6 plays important role in the transmission of thermal sensation under both physiological and pathological conditions in mice. Additionally, there was a marginal effect on the transmission of mechanical sensation only under pathological conditions.

### 3.3 TMC6 in DRG modulates heat sensitivity in mice

**FIGURE 3:**
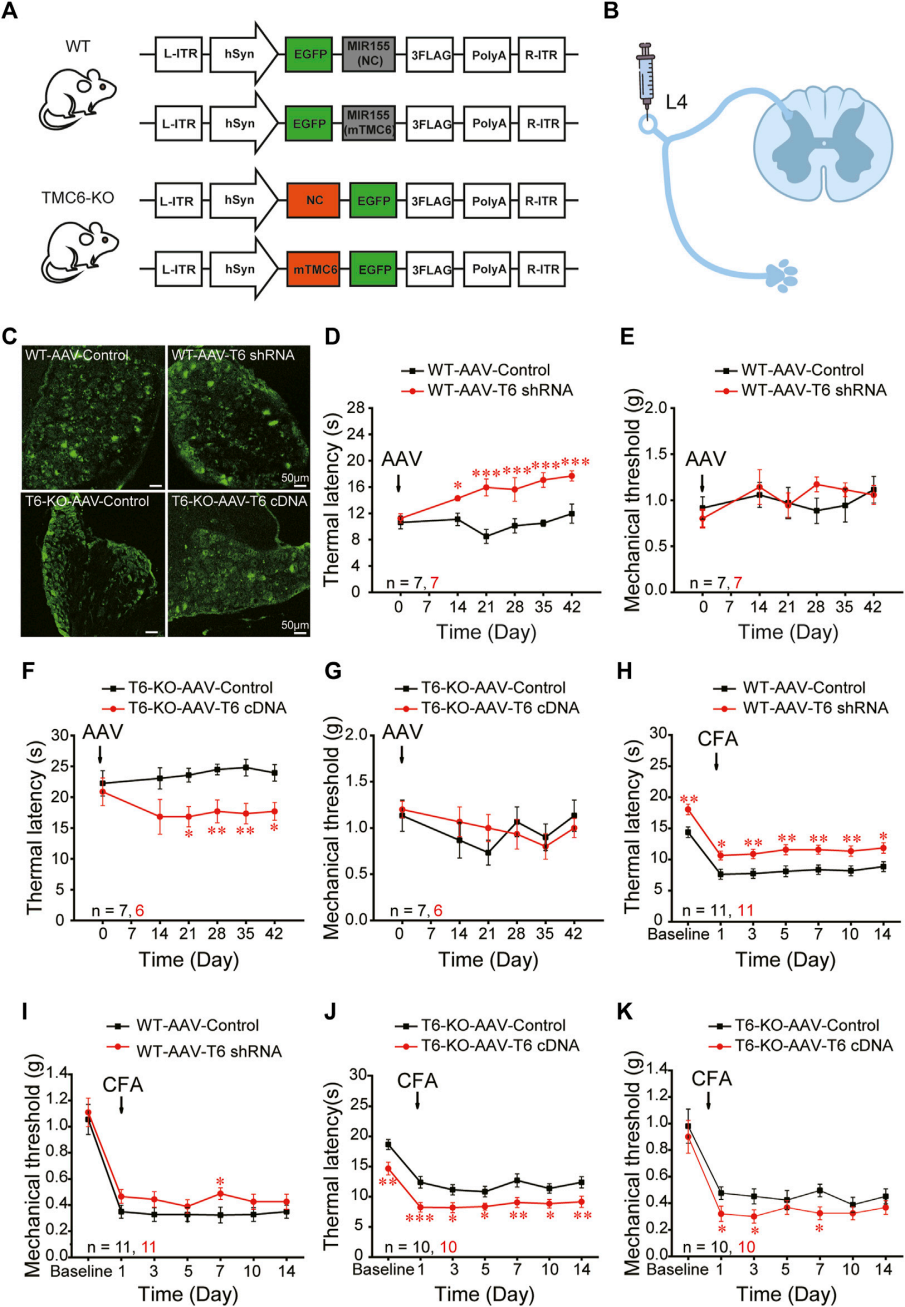
TMC6 in DRG modulates heat sensitivity of mice. **(A)** Schematic diagram depicts the design of viruses. TMC6 knock-down with AAV9-mTMC6-shRNA in male WT mice; TMC6 gene overexpression with AAV9-mTMC6-cDNA in TMC6-KO male mice and their respective control. **(B)** Schematic diagram depicts the sites (L4 DRG) for the injections of designed viruses in mice. **(C)** Confirmation of EGFP (green) expression in L4 DRG of the WT male mice received AAV9-mTMC6-shRNA virus and TMC6-KO male mice received AAV9-mTMC6-cDNA virus viral infection. Scale bars, 50 μm. **(D–G)** The Hargreaves test **(D,F)** and Von Frey test **(E,G)** were performed after injection the AAV9-mTMC6-shRNA and AAV9-mTMC6-cDNA virus. **(H–K)** The comparison of chronic inflammatory pain induced by CFA injection between TMC6-KO male mice and their WT littermates. Hargreaves **(H,J)** and Von Frey tests **(I,K)** were used to assay the chronic pain behaviours. Data are shown as mean ± SEM. two-way repeated ANOVA with the Tukey post-test was used for comparison of behavioural measurements within the corresponding time points: *p < 0.05, **p < 0.01, ***p < 0.001.

To explore whether the altered sensation phenotype of TMC6*-*KO mice was due to the deletion of TMC6 in the peripheral sensory ganglion, we constructed the vectors of AAV9-viruses carrying either EGFP-tagged TMC6-shRNA or TMC6-cDNA to knockdown TMC6 in the DRG of WT male mice or to re-express TMC6 in the DRG of TMC6-KO male mice (Figure 3A). The AAV9-viruses (2 μL, 5 × 10^12^ v. g./mL) were injected into the exposed right L4 DRG of male mice (Figure 3B). As shown in Figure 3C, EGFP fluorescence was readily detectable 4 weeks after virus injection. TMC6 mRNA expression was significantly down- or upregulated by virus injection in the L4 DRG of WT or TMC6-KO male mice, respectively, as determined by real-time quantitative PCR detecting (Supplementary Figure S3). Basal sensitivities to mechanical and heat stimuli were measured per week after virus injection 2–6 weeks. The results demonstrated that WT male mice injected with AAV9-mTMC6-shRNA virus exhibited significantly prolonged thermal withdrawal latencies starting from the third week after virus injection, in comparison to WT male mice injected with the control virus (Figure 3D), whereas there was no significant change observed in mechanical sensitivity (Figure 3E). In TMC6-KO male mice, injection of AAV9-TMC6-cDNA viruses significantly increased the thermal sensitivity (lower thermal withdrawal latency) starting from the third week of virus injection (Figure 3F). AAV9-TMC6-cDNA virus did not significantly change the mechanical sensitivity of TMC6-KO male mice (Figure 3G).

Next, we explored the effect of interfering TMC6 in DRG on chronic pain. During the third week of maintaining stable viral expression in animals, we induced chronic inflammatory pain. In the CFA models, WT mice that received AAV9-mTMC6-shRNA virus injection displayed alleviating thermal hyperalgesia and significantly higher thermal withdrawal latencies at all tested time points throughout the 2 weeks (Figure 3H). However, WT mice that received AAV9-mTMC6-shRNA virus injection displayed only marginal alleviation of mechanical hyperalgesia and a significantly higher mechanical threshold on the 7th day (Figure 3I). As expected, TMC6-KO mice that received AAV9-TMC6-cDNA virus administration exhibited decreased withdrawal latencies in peripheral heat pain behaviour at all tested time points throughout the 2 weeks, after CFA injection (Figure 3J). Nevertheless, mechanical sensation was only decreased on the 1st, 3rd and 7th days (Figure 3K). These findings suggest that peripheral TMC6 in the DRG plays a more significant role in thermal sensation compared to mechanical sensation.

### 3.4 TMC6 deletion hyperpolarizes resting membrane potential and inhibits excitability of DRG neurons in mice

**FIGURE 4:**
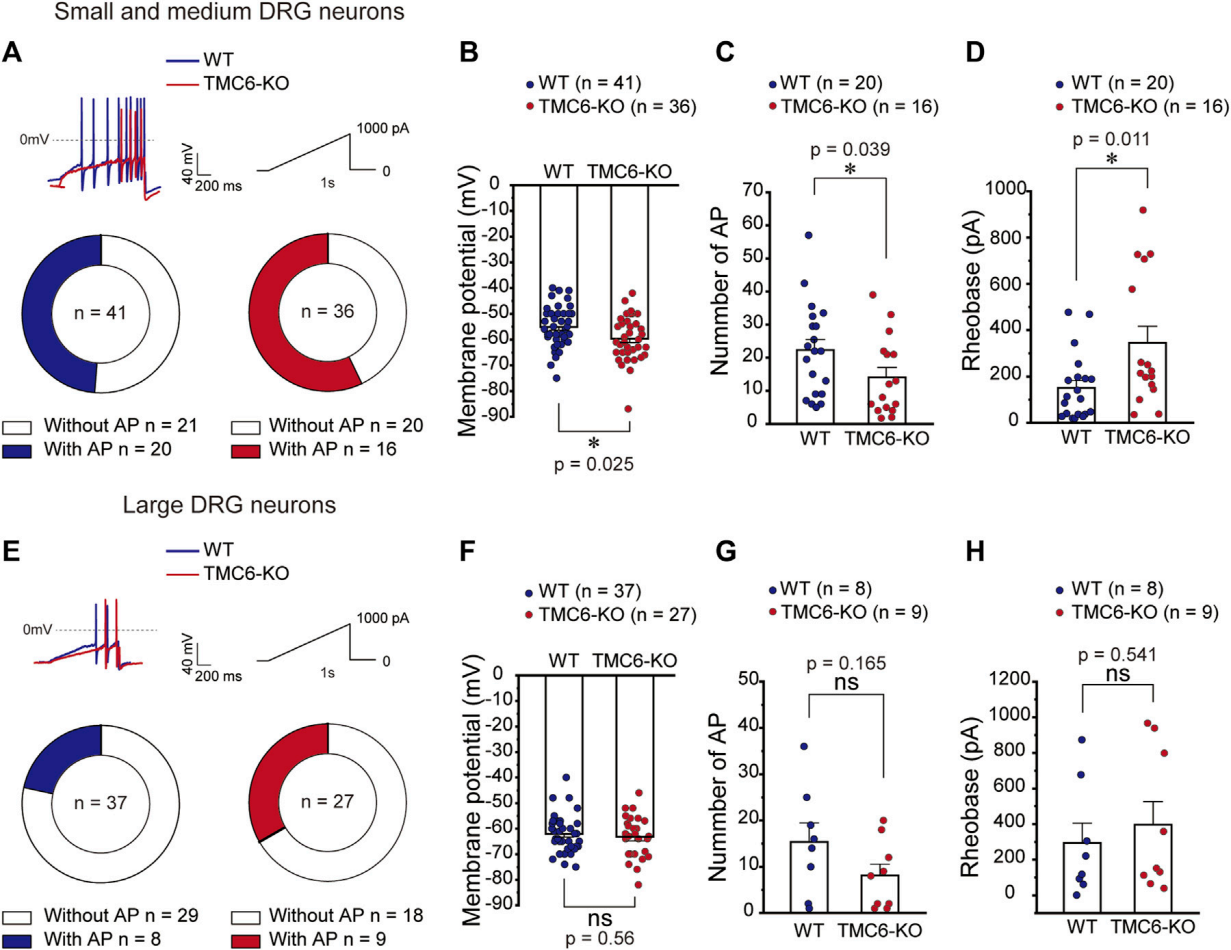
TMC6 affects the excitability of DRG neurons. **(A,E)** Upper panel: Representative traces of AP currents stimulated with a ramp current from 0 to 1,000 pA (1s) in small-medium DRG neurons **(A)** and large DRG neurons **(E)**. **(A,E)** Below panel: The proportion of neurons showing AP responding to the ramp current stimulation of WT and TMC6-KO mice in small-medium **(A)** and large DRG neurons **(E)**, respectively. **(B,F)** The comparison of membrane potential of WT and TMC6-KO mice in small-medium and large DRG neurons, respectively. **(C,G)** The number of AP evoked by the ramp current stimulation in small-medium and large DRG neurons from WT and TMC6-KO mice, respectively. **(D,H)** The rheobase of AP evoked by the ramp current stimulation in small-medium and large DRG neurons from WT and TMC6-KO mice, respectively. Data are shown as mean ± SEM, Pearson’s chi-square test **(A,E)**, *t*-test **(B,F)**, Mann-Whitney U test **(C, D, G,** and **H)** *p < 0.05, ns: no significant.

To explore the mechanism that TMC6 deletion in DRG neurons decreases thermal sensitivity in mice, we first tested the RMP and excitability of small-medium (Figures 4A–D) and large (Figures 4E–H) DRG neurons in both male and female WT and TMC6-KO mice. A ramp current clamp protocol was used to evoke action potential from dissociated WT and TMC6-KO DRG neurons (Figure 4A, upper panel). In small and medium DRG neurons, the proportion of neurons in which showed evoked action potentials (AP) firing had no significant difference between TMC6-KO and WT DRG neurons (Figure 4A, lower panel). However, TMC6-KO DRG neurons exhibited a hyperpolarized resting membrane potential compared with WT neurons (Figure 4B). The number of evoked AP in TMC6-KO DRG neurons was significantly lower than that in WT DRG neurons (Figure 4C). Meanwhile, the injected current rheobase of AP was significantly higher in TMC6-KO DRG neurons (Figure 4D). In large DRG neurons, there were no significant differences in the proportion of neurons showing evoked action potential, RMP, AP numbers and current rheobase between WT and TMC6-KO large DRG neurons (Figures 4E–H). Taken together, these results suggest that TMC6 deletion reduces the excitability of small to medium DRG neurons, which may lead to the low sensitivity of TMC6-KO mice to peripheral thermal stimuli.

### 3.5 TMC6 modulates M channel function

**FIGURE 5:**
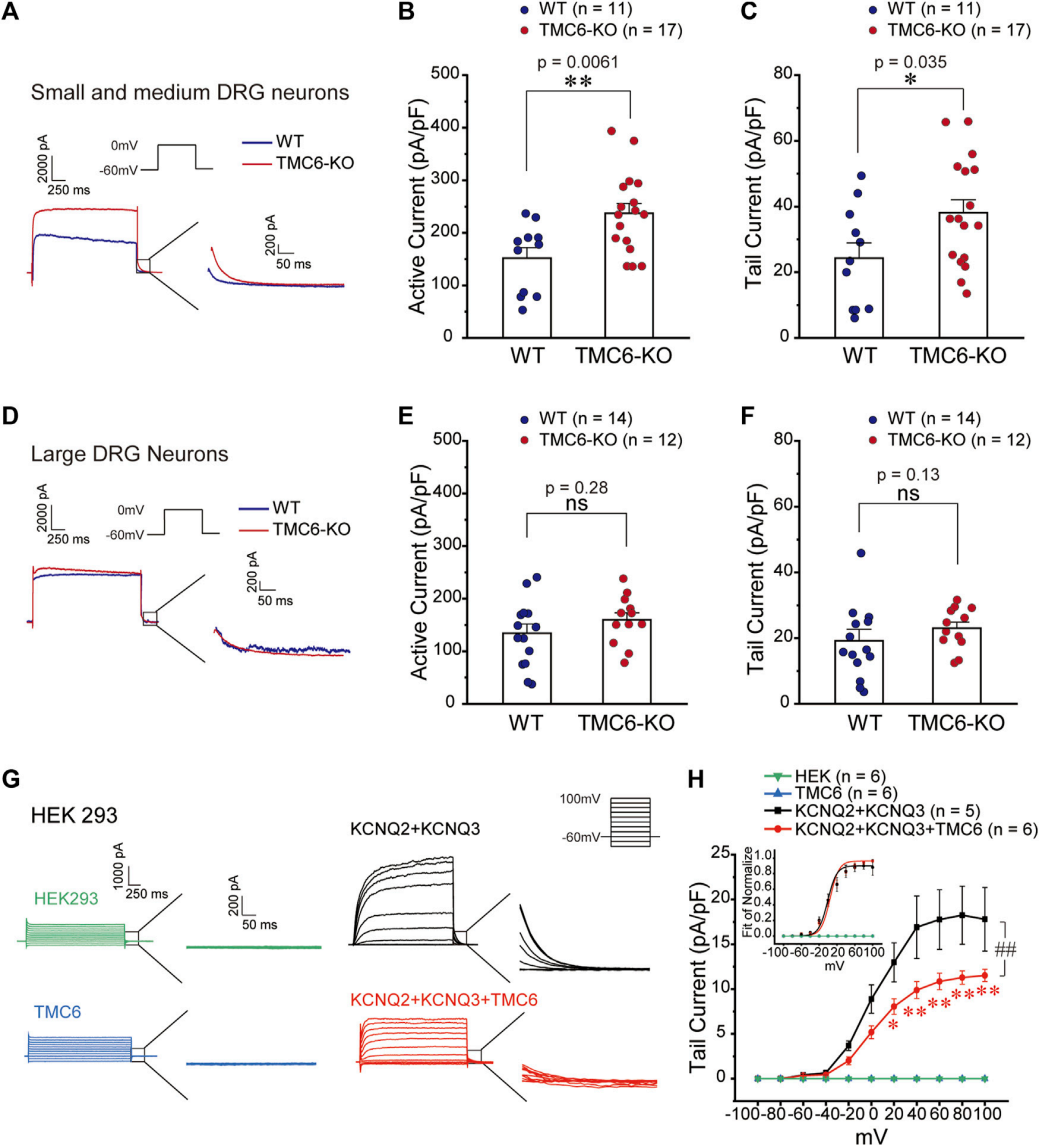
TMC6 inhibits the M current. **(A,D)** Representative current traces of M current induced by the indicated single step protocol (upper inset) from small-medium **(A)** and large **(D)** DRG neurons. Enlarged tail currents (right inset). **(B,C,E,** and **F)** Statistic comparisons of the active current **(B,E)** and tail current **(C,F)** of M channel between TMC6-KO and WT DRG neurons in mice. Statistic result of the active current of M channel in small-medium **(B)** and large **(E)** DRG neurons in mice. Statistic results of the tail current of M channel in small-medium **(C)** and large **(F)** DRG neurons between TMC6-KO and WT. **(G)** Representative current traces of M current induced by the indicated multiple-step protocol (upper inset) in HEK293 cells expressing KCNQ2/3 with or without TMC6. HEK293 cells and TMC6 alone were used as controls. Enlarged tail currents (right inset). **(H)** The tail current densities of the M current were compared among HEK293 cells expressing KCNQ2/3 with or without TMC6, as well as HEK293 cells and TMC6 alone. The normalized G-V curves of the M current recorded from HEK293 cells were fitted using the Boltzmann equation (upper inset). Data were shown as mean ± SEM, *t*-test **(B,C,** and **E)**, Mann-Whitney U test **(F)**, and Two-way repeated ANOVA **(H)**, *p < 0.05, ** or ##*p* < 0.01, ns: no significant.

M channels are the major molecules that influence the cell membrane potential and excitation in DRG neurons (Du et al., 2014). We investigated the effect of TMC6 deletion on M current in small-medium (Figures 5A–C) and large (Figures 5D–F) DRG neurons in both male and female mice. Using the voltage protocol shown at the top of the Figure 5A, M currents were recorded from dissociated and cultured DRG neurons from WT and TMC6*-*KO mice. In small and medium DRG neurons, the activation currents from −60 mV to 0 mV and tail currents from 0 mV to −60 mV were analysed. As shown in Figures 5B, C, the amplitude of both the activation currents and tail currents were significantly increased in small to medium TMC6*-*KO DRG neurons in comparison to WT neurons. However, amplitudes of neither activation nor tail currents were exhibited significant differences in large DRG neurons from TMC6-KO and WT mice (Figures 5D–F).

To further investigate the effect of TMC6 on M channel, we co-transfected the M channel components KCNQ2 and KCNQ3 with or without TMC6 into HEK293 cells, using HEK293 cells and TMC6 alone as controls (Figures 5G, H). Figure 5G displays the current traces, recorded using a voltage-step protocol with a range from −100 mV to 100 mV, a 20 mV interval, and a holding potential of −60 mV. The tail currents are measured as indicated in Figure 5G (inset). The tail current of M current induced by voltage from 20 to 100 mV was significantly inhibited by co-expressing TMC6 with the channel (Figure 5H). The G-V curve plotted from the tail currents of M channel was not affected by co-expressing TMC6 in HEK293 cells (Figure 5H, inset). Taken together, these findings provide evidence that TMC6 is capable of inhibiting M channel current, but does not have an impact on its voltage sensitivity.

### 3.6 Mechanism of TMC6 influences M channel in DRG neurons

**FIGURE 6:**
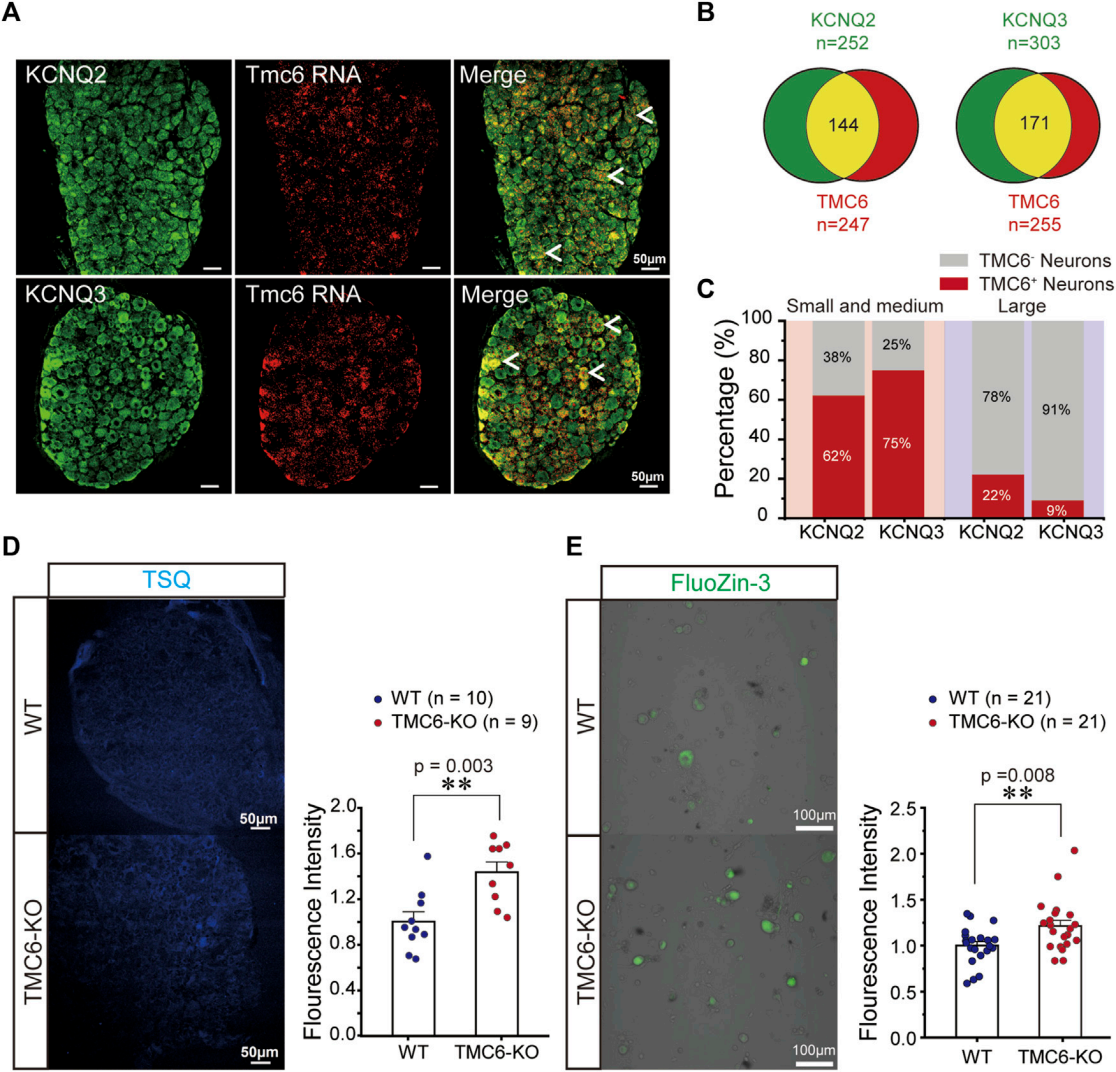
Mechanism of TMC6 modulating M currents in DRG neurons. **(A)** DRG slices were stained for TMC6 mRNA using RNAscope probe (red) and antibodies against KCNQ2 or KCNQ3 (green) protein were used for immunofluorescence in DRG neurons. Arrows indicate co-localization of TMC6 mRNA with KCNQ2 or KCNQ3 protein. Scale bars, 50 μm. **(B)** The pie diagram indicates the distribution of DRG neurons expressing KCNQ2 or KCNQ3 (green), and the number of DRG neurons expressing TMC6 (red). The co-expressing of the KCNQ2 or KCNQ3 protein with TMC6 mRNA is shown as the yellow area. The analyse was obtained from 4 DRG slices. **(C)** The percentage of TMC6-positive neurons among KCNQ2-positive and KCNQ3-positive in small-medium and large DRG neurons respectively. **(D)** DRG slices from TMC6-KO and WT mice were stained with the fluorescent Zn^2+^ indicator, TSQ (left). A comparison was made between TMC6-KO and WT DRG slices in terms of TSQ fluorescent intensity (right). Analysis was performed on 10 or 9 slices obtained from 3 animals, WT: *n* = 10, TMC6-KO: *n* = 9. **(E)** Cultured DRG neurons were incubated with FluoZin-3, a fluorescent zinc indicator (left). The comparison of FluoZin-3 fluorescent intensity between TMC6-KO and WT DRG neurons. Analysis was performed on 21 slices obtained from 4 animals, WT or TMC6-KO: *n* = 21. Data are presented as mean ± SEM; *t*-test (**D,E**; right), **p < 0.01.

To elucidate the mechanism of TMC6 inhibiting M channels in DRG neurons, we first investigated whether TMC6 was co-expressed with KCNQ2 and KCNQ3 in DRG neurons. The combination of RNAscope *in situ* hybridization targeting TMC6 mRNA and immunostaining for KCNQ2 and KCNQ3 proteins in the DRG revealed a significant overlap between TMC6 mRNA and the KCNQ2 and KCNQ3 proteins in DRG neurons, as depicted in Figure 6A. The number of neurons marked with KCNQ2, KCNQ3, and TMC6 were analysed 4 DRG slices (Figure 6B). Among the small to medium-sized DRG neurons, the proportion of TMC6-positive neurons was found to be as high as 62% of KCNQ2-positive neurons and 75% of KCNQ3-positive neurons. However, in the case of large DRG neurons, TMC6-positive neurons accounted for only 22% of KCNQ2-positive neurons and 9% of KCNQ3-positive neurons (Figure 6C). The co-expression of TMC6 with KCNQ2 or KCNQ3 provides the possibility of modulating the M channel in the small and medium DRG neurons.

The above electrophysiological experiments indicated that TMC6 deletion increased the amplitude of the M current in the small and medium DRG neurons. Notably, a previous study demonstrated direct activation of the M channel by intracellular zinc (Gao et al., 2017). TMC6 (EVER6) and TMC8 (EVER6) were reported forming complexes with Zn transporter 1 (ZnT-1) in the endoplasmic reticulum (ER), which is involved in the regulation of cellular Zn^2+^ trafficking in the cell (Lazarczyk et al., 2008). Most likely, the ER-residing ZnT-1/EVER complex is involved in the transfer of Zn^2+^ from the cytoplasm into the ER lumen, interfering with the spreading of the zinc wave and the function of Zn^2+^ as a second messenger (Lazarczyk and Favre, 2008). We hypothesized that TMC6 deletion in DRG neurons might increase the concentration of free cytoplasmic zinc and affect M channel function. Staining of DRG slices from WT and TMC6-KO mice with N-(6-methoxy-8-quinolyl)-p-toluene-sulfonamide (TSQ) (Kiyohara et al., 2021), a Zn^2+^ fluorophore, revealed higher fluorescence values in DRG tissue from TMC6-KO mice than in that of WT mice (Figure 6D). Then we dissociated and cultured DRG neurons from WT and TMC6-KO mice, and incubated the cultured DRG neurons with another Zn^2+^ probe, FluoZin-3 (Han et al., 2018), for 30 min. As shown in Figure 6E, DRG neurons in TMC6-KO mice showed a significant increase in Zn^2+^ fluorescence compared with those in WT mice. These results suggest that TMC6 deletion increases free zinc levels in the cytoplasm of DRG neurons, and the upregulation of cytoplasmic free zinc may activate M channels.

## 4 Discussion

**FIGURE 7:**
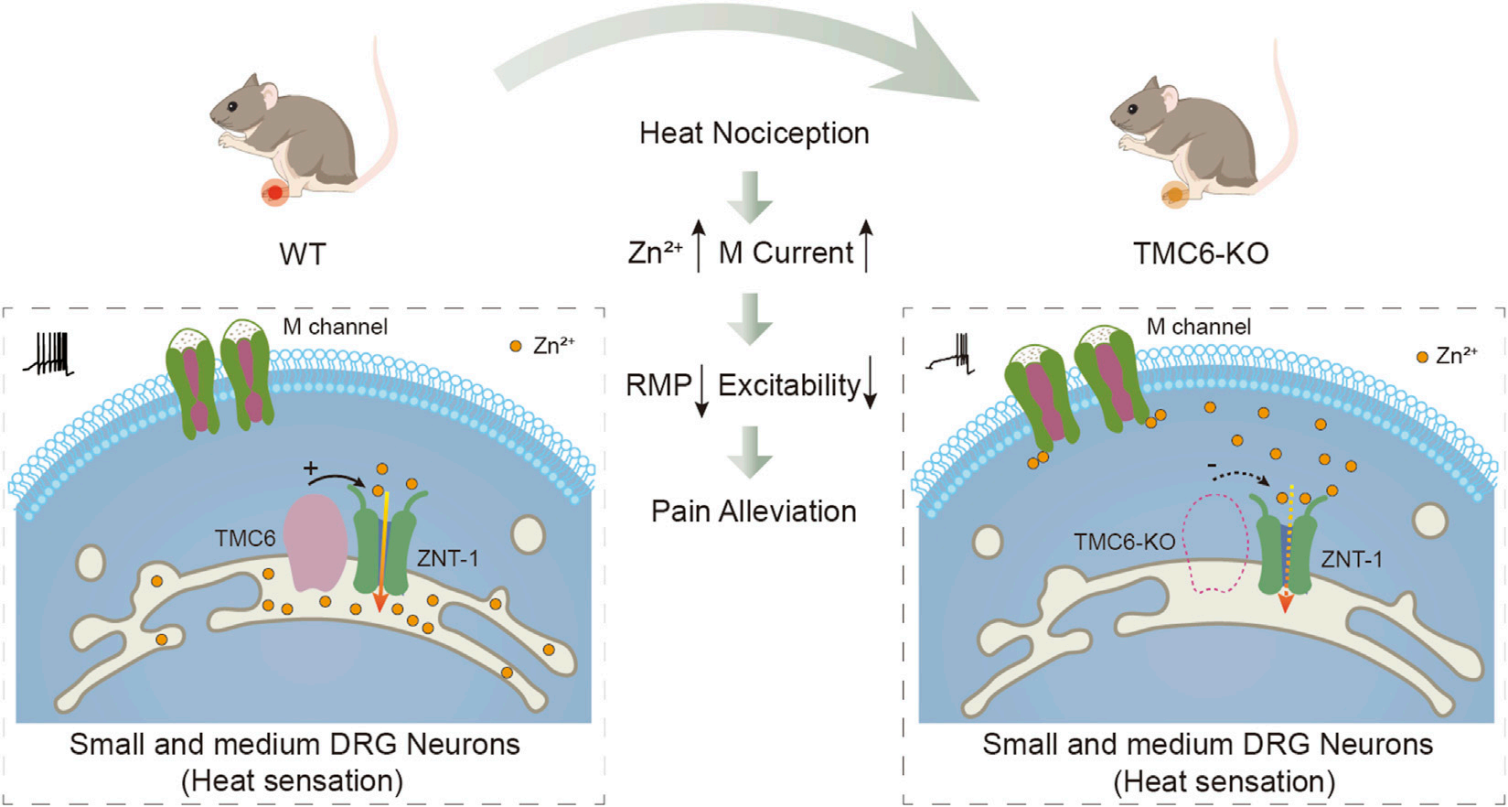
Hypothesis diagram illustrating the potential role of TMC6 in peripheral heat sensation. TMC6 exhibits high expression levels in small and medium-sized dorsal root ganglion (DRG) neurons. Deletion of TMC6 may influence ZNT-1, resulting in an elevation of free zinc concentration, subsequently activating the M channel. Activation of the M channel results in hyperpolarization of the resting membrane potential (RMP) in DRG neurons, and a decrease in neuronal excitability. Consequently, the heat sensitivity of TMC6 knockout mice is reduced.

In this study, we proposed that TMC6 in primary sensory neurons plays an important role in thermal sensation by modulating the M channel. Our findings support the below hypotheses: 1) TMC6 mRNA is abundantly expressed in in small and medium DRG neurons; 2) Deletion of TMC6 in DRG neurons *in vivo* results in marked blunting of physiological heat sensation and heat hyperalgesia in chronic pathological pain; 3) TMC6 deletion increases the M current amplitude in the small and medium DRG neurons but does not affect the activating curve of the channel; 4) TMC6 inhibits heterogeneously expressed M channel currents in HEK293 cells; 5) TMC6 deletion increases free Zn^2+^ in DRG neurons which is an effective M channel activator. According to these evidences, we depicted a hypothesis working model of TMC6 involved in peripheral heat nociception as shown in Figure 7.

The mammalian transmembrane channel-like (TMC) protein family contains eight members, from TMC1–TMC8. This family can be grouped into three subfamilies: A (TMC1–TMC3), B (TMC5 and TMC6), and C (TMC4, TMC7, and TMC8) (Keresztes et al., 2003). Notably, the high degree of similarity between the corresponding human and murine TMCs suggests a conserved function for these proteins. It is known that TMC1 and TMC2 are expressed in cochlear hair cells and are crucial for auditory sensation (Kurima et al., 2015; Pan et al., 2018). They were recently shown to be the long sought-after pore-forming subunits of the hair cell transduction channel (Pan et al., 2013; Pan et al., 2018; Jia et al., 2020; Tang et al., 2020). TMC6 and TMC8 have been reported to contribute to epidermodysplasia verruciformis, and predisposition to HPV (Ramoz et al., 2002; de Jong et al., 2018a; de Jong et al., 2018b; Wu et al., 2020). However, the relationship between TMC and the development of peripheral chronic pain remains unclear. The present investigation provides evidence for a previously unknown function of TMC6 in primary sensory neurons. This study represents the first demonstration of the crucial role played by TMC6 in the development of thermal hyperalgesia.

In terms of mechanism, we demonstrated that TMC6 may affect peripheral sensations by modulating the M channel. M channel, constituted by KCNQ2/3, regulates excitability in a variety of central and peripheral neurons (Brown, 1988; Marri and on, 1997). Ample studies suggest that M channel plays a key role in regulating excitability in nociceptors and may therefore present a novel therapeutic target for the treatment of pain (Delmas and Brown, 2005; Zhang et al., 2011; Du et al., 2014). Our results show that TMC6 deletion increases the zinc concentration in DRG neurons. Zinc is an essential trace element required for enzymatic activity and maintaining the conformation of many transcription factors (Prasad, 1995; Vallee and Auld, 1995; Kambe et al., 2015). ER has been demonstrated to be an important organelle where zinc is stored and from where, upon stimulation, a reservoir of Zn^2+^ can be rapidly released into the cytoplasm and subsequently passed to the nucleus; this sequence has been designated a “zinc wave” (Yamasaki et al., 2007). Multiple studies have demonstrated that TMC6 (EVER6) and TMC8 (EVER8) are localised in the ER and form complexes with ZnT-1 (Lazarczyk et al., 2008; Lazarczyk and Favre, 2008). TMC8 is required to lower cytosolic Zn^2+^ concentrations by modulating ZnT-1 (Siri et al., 2014). Recently, zinc has been defined as an intracellular second messenger capable of transducing extracellular stimuli into intracellular signalling events, and zinc homeostasis is tightly regulated (Frederickson et al., 2000; Kambe et al., 2015; Bafaro et al., 2017; Gammoh and Rink, 2017). Zinc activates M channels by reducing their dependence on phosphatidylinositol 4,5-bisphosphate (PIP_2_) (Gao et al., 2017). Therefore, we hypothesize that one possible mechanism for potentiating the of M channel current in TMC6-KO DRG neurons is the increase in cytoplasmic zinc. But we acknowledge that this hypothesized mechanism may be only one possibility on how TMC6 deletion resulting in enhancement of M channel function.

Interestingly, our results reveal that the impact of TMC6 knockout is primarily observed in sensitivity to heat, rather than mechanical stimuli. The dorsal root ganglion (DRG) tissue comprises various neuron types traditionally categorized based on the presence or absence of myelin and the size of the neuron bodies. Nociception is transmitted by both myelinated Aδ fibers with medium-sized and unmyelinated C fibers with small cell bodies, while mechanoreception is facilitated by myelinated Aβ fibers with large cell bodies. Notably, our investigation revealed a predominant co-expression of TMC6 and KCNQ2/3 in medium and small-sized DRG neurons, which are also known to abundantly express the temperature-sensitive channel transient receptor potential vanilloid 1 (TRPV1) (Bamps et al., 2021). Importantly, we also investigated the impact of TMC6 deletion on the expression and function of the TRPV1 channel in DRG neurons (Supplementary Figure S4). Western blot analysis shows that there is no significant difference between the expression of TRPV1 protein in the DRG of WT and TMC6-KO mice (Supplementary Figure S4A). The agonist of TRPV1 channel capsaicin (1 μM) was used to test the TRPV1 function in WT and TMC6 KO DRG neurons. It is shown that function of TRPV1, including the positive response rate and current amplitude, has no significant difference between DRG neurons of WT and TMC6-KO mice (Supplementary Figure S4B). We hypotheses that the specific impact of TMC6 knockout on heat sensation stems from the following reasons: ①TMC6 is preferentially expressed in small and medium-sized DRG neurons (primarily involved in nociception including heat transmission), with less expression in large DRG neurons (primarily involved in mechanoreception) (Figures 1A–C). ②TMC6 and KCNQ2/3 were predominantly co-expressed in medium and small-sized DRG neurons (Figures 6A–C). ③The electrophysiological recordings have further validated that the M channel function was significantly upregulated in small to medium-sized DRG neurons of TMC6-KO mice compared to WT mice, but not in large DRG neurons (Figures 5A–F). Noticeably, we did not test if TMC6 itself is heat sensitive, which also could be another reason of its primarily affecting heat but not mechanosensitivity.

Chronic pain is a complex condition with limited treatment options. Additionally, our findings demonstrate that TMC6 deletion in DRG neurons remarkably alleviates heat hyperalgesia in both chronic pain inflammatory and neuropathic pain models, but only marginally alleviates on mechanical allodynia. These findings suggest that TMC6 may be a potential therapeutic target for chronic pain.

## Data Availability

The original contributions presented in the study are included in the article/Supplementary Material, further inquiries can be directed to the corresponding author.
