# Supplementary material for: The transmembrane channel-like 6 (TMC6) in primary sensory neurons involving thermal sensation via modulating M channels
Source: Front Pharmacol. 2024 Feb 19;15:1330167. doi: 10.3389/fphar.2024.1330167 (PMC10909837; doi:10.3389/fphar.2024.1330167)
Supplement: Supplementary file 1 [file DataSheet1.docx]

**Supplementary figures**
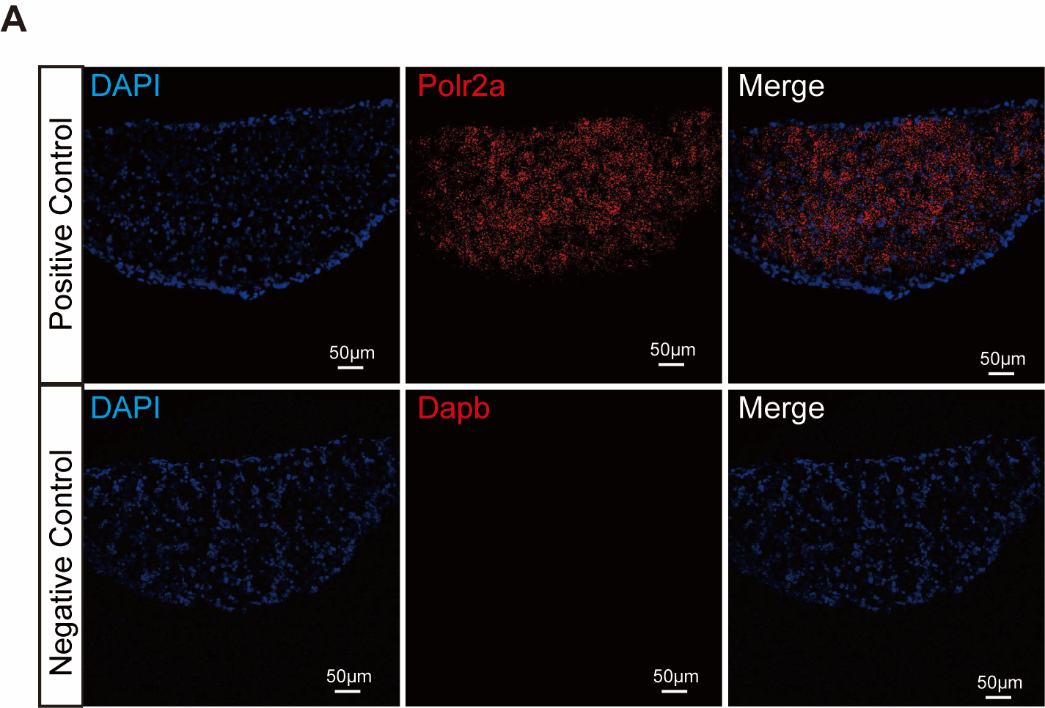


**Supplementary Figure S1** The positive (Polr2a) and negative (Dapb) control of RNAscope in WT DRG slices.


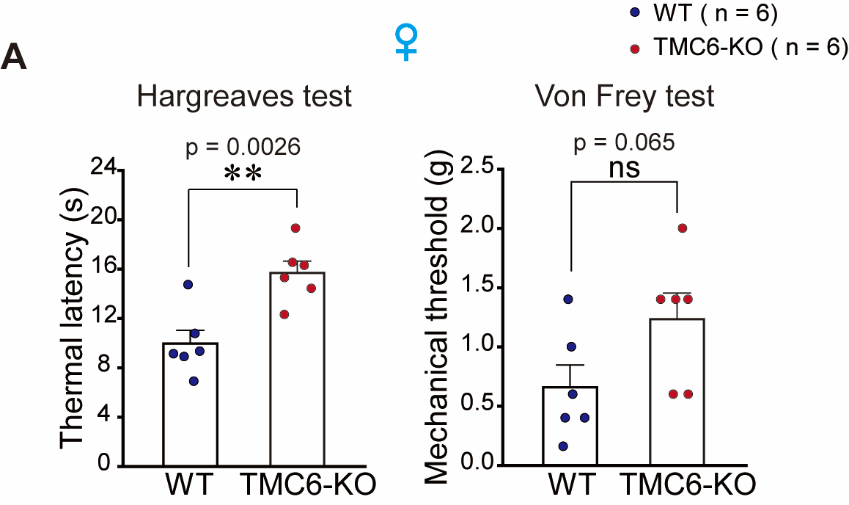


**Supplementary Figure S2** The thermal and mechanical sensitivities measured using Hargreaves and Von Frey tests in female WT and TMC6-KO mice.

Data are shown as the mean ± SEM, *t*-test **(A left)** and Mann-Whitney U test **(A right).** **p < 0.01, ns: not significant.


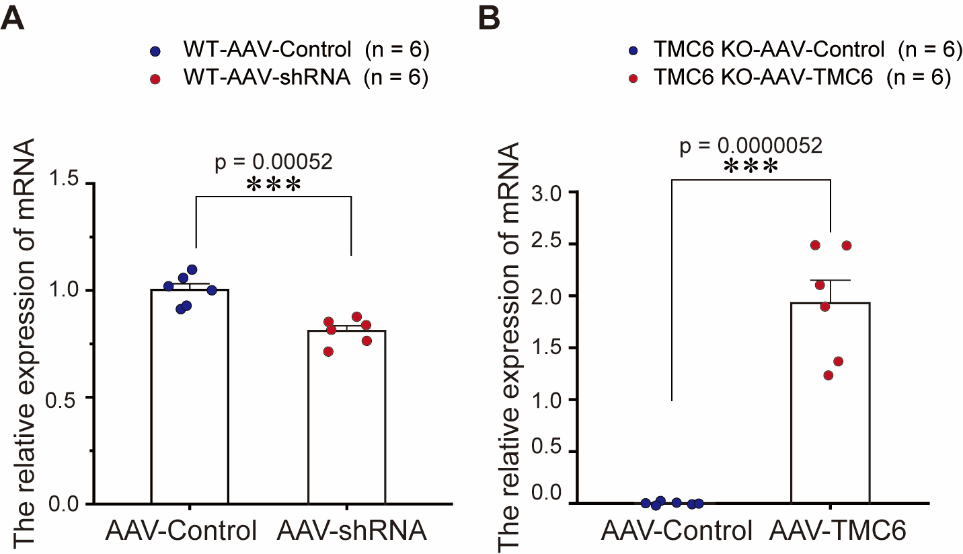


**Supplementary Figure S3** TMC6 mRNA expression levels examined by real-time quantitative PCR after 8 weeks of corresponding virus injection in the L4 DRG.

The mRNA expression of TMC6 in WT mice injected with AAV9-mTMC6-shRNA **(A)** and in TMC6-KO mice injected with AAV9-mTMC6-cDNA viral infection **(B)**, relative to their corresponding AAV9-Control.

Data are shown as the mean ± SEM, *t*-test **(A and B)**, ***p < 0.001.


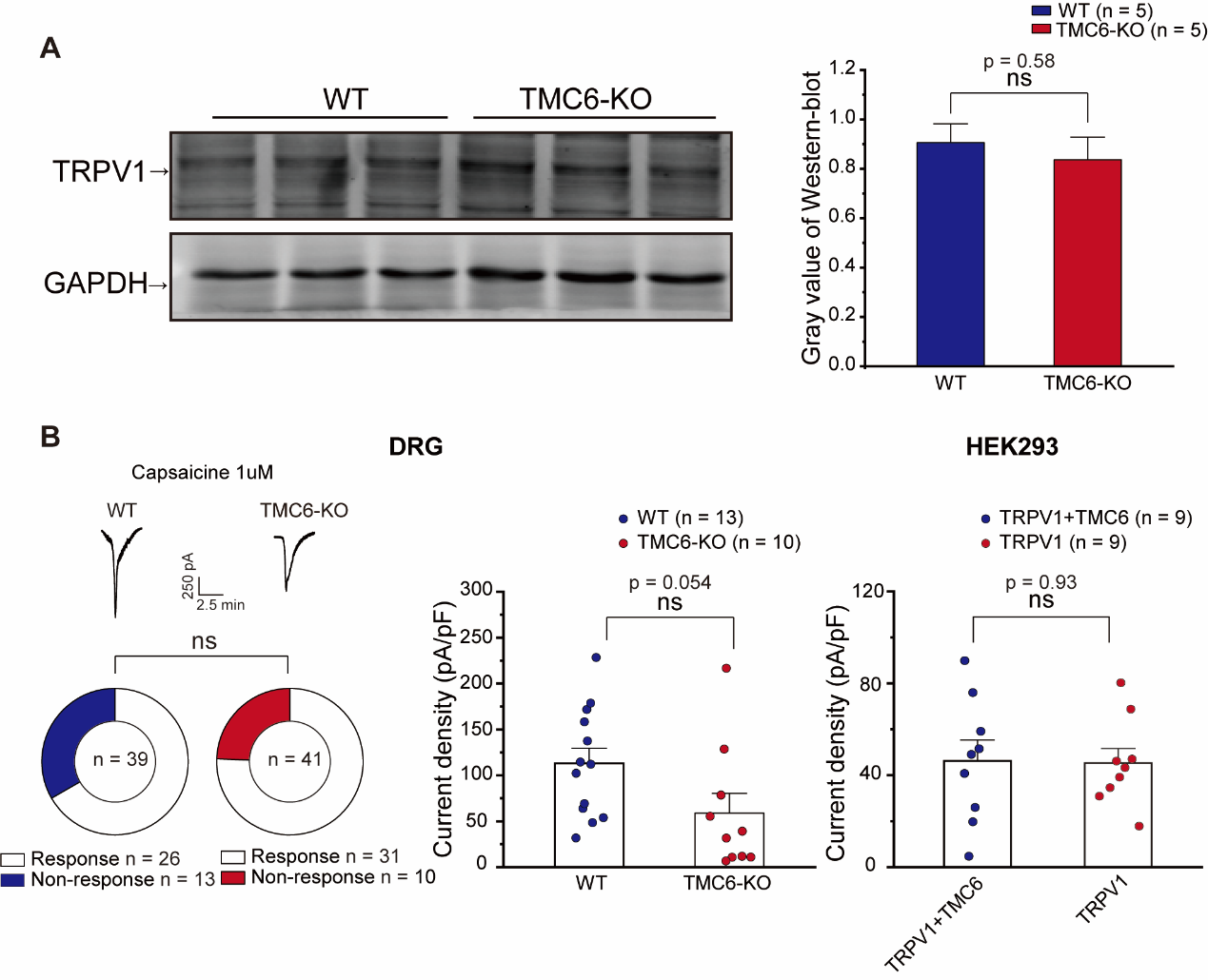


**Supplementary Figure S4** The impact of TMC6 deletion on the expression and function of TRPV1 channel in the DRG neurons.

**(A)** Western blot of TRPV1 protein in DRG from WT and TMC6-KO mice and the quantitative analysis.

**(B)** Comparison currents of TRPV1 induced by agonist Capsaicine (1 μM) between WT and TMC6-KO DRG neurons and HEK293 cells. The traces of TRPV1 currents were recorded holding at −60mV potential (left top panel). The proportion of Capsaicine-responding and non-responding DRG neurons between WT and TMC6-KO (left bottom panel). The plots were TRPV1 channel current densities induced by Capsaicine in WT and TMC6-KO DRG neurons and HEK293 cell transfected plasmids.

Date show mean ± SEM, **(B left panel)** Pearson’s chi-square test, **(A right panel, B middle and right panels)** *t*-test, ns: no significant.
